# Incidence and Demographic, Clinical, and Occupational Patterns of Dental Caries and Gingivitis in Workers: A Retrospective Descriptive Cohort Study

**DOI:** 10.3390/jcm15197650

**Published:** 2026-10-02

**Authors:** Fiorella E. Zuzunaga-Montoya, Jhosmer Ballena-Caicedo, Carmen Inés Gutierrez De Carrillo, Víctor Juan Vera-Ponce, Oriana Rivera-Lozada, Olger E. Bernal-Santos, Mario J. Valladares-Garrido

**Affiliations:** 1 Escuela de Medicina Humana, Universidad Continental, Lima 15306, Peru; fiorella.zuzunaga@untrm.edu.pe; 2 Facultad de Medicina (FAMED), Universidad Nacional Toribio Rodríguez de Mendoza de Amazonas (UNTRM), Chachapoyas 01001, Peru; 7330178022@untrm.edu.pe (J.B.-C.); carmen.gutierrez@untrm.edu.pe (C.I.G.D.C.); victor.vera@untrm.edu.pe (V.J.V.-P.); 3 Escuela de Medicina Humana, Universidad Señor de Sipán, Chiclayo 14001, Peru; riveraoriana@uss.edu.pe (O.R.-L.); bsantosolgerema@uss.edu.pe (O.E.B.-S.); 4 EpiHealth Research Center for Epidemiology and Public Health, Lima 15001, Peru; 5 Asociación Científica de Estudiantes de Medicina, Universidad Señor de Sipán, Chiclayo 14001, Peru; 6 Oficina de Inteligencia Sanitaria, Red Prestacional Lambayeque, EsSalud, Chiclayo 14001, Peru

**Keywords:** dental caries, gingivitis, occupational health, cohort studies, incidence, periodontal diseases, occupational dentistry

## Abstract

**Objective:** To estimate the incidence of clinically detected dental caries and gingivitis in a cohort of workers undergoing periodic occupational evaluations, and to describe crude demographic, clinical, behavioral, and occupational differences in these events. **Methods:** A retrospective descriptive cohort study with secondary analysis of occupational clinical records. We included 12,049 workers with at least two dental evaluations separated by a minimum of one year. Incidence rates were estimated per 1000 person-years with 95% confidence intervals (95% CIs). Crude hazard ratios (HRs) were calculated using univariate Cox models as descriptive time-to-event contrasts. Continuous variables were analyzed in 5-unit increments, and age- and sex-standardized rates were estimated in supplementary analyses. **Results:** After correcting person-time to person-years, the incidence of caries was 14.86 per 1000 person-years (95% CI 13.55–16.27), and the incidence of gingivitis was 4.67 per 1000 person-years (95% CI 3.95–5.49). Male workers showed higher observed incidence of caries than female workers (crude HR 1.81, 95% CI 1.32–2.48). Manual/physical workers also showed higher observed incidence of caries than office/administrative workers, whereas occupational differences in gingivitis were smaller after age and sex standardization. A graded descriptive pattern was observed with years of occupational exposure, with crude HRs of 3.61 for caries and 3.11 for gingivitis among workers with ≥10 years of exposure compared with those with <1 year. Baseline glucose showed a positive crude time-to-event contrast with caries (HR 1.04, 95% CI 1.02–1.06), and systolic blood pressure showed a positive crude time-to-event contrast with gingivitis (HR 1.07, 95% CI 1.01–1.13). **Conclusions:** Incident clinically detected caries and gingivitis were uncommon but showed crude differences across sex, occupational category, job tenure, and selected cardiometabolic markers. These findings may inform preventive attention and referral strategies in occupational dental surveillance, although the crude estimates should not be interpreted as causal effects.

## 1. Introduction

Dental caries and gingivitis are two of the most common oral conditions and contribute significantly to the global burden of disease, with functional, psychosocial, and economic repercussions. The most recent international reports estimate that around 3.5 billion people have some form of oral disease; untreated caries in permanent teeth affect approximately 2.5 billion individuals, and severe periodontal disease affects around 1 billion. These figures show that oral health continues to be a critical component of noncommunicable diseases and health equity [1].

Within this context, the working population is a group of particular interest. Even though many companies implement periodic occupational evaluations that include clinical screenings and health promotion, workers face exposures and conditions in the work environment, such as shift work, time demands, stress, and differences by occupation type, that can affect hygiene behaviors and the timely use of dental services. The literature suggests differences in oral health associated with occupation, and some studies have described worse oral outcomes in shift work contexts. In addition, the interval between dental checkups in the workplace can influence clinical outcomes and costs, although the optimal schedule is not fully defined [2,3,4,5].

Occupational conditions may shape oral health through behavioral, organizational, and biological pathways. Long or irregular schedules can reduce opportunities for toothbrushing, preventive dental visits, and timely treatment; workplace diet patterns may increase exposure to fermentable carbohydrates; and psychosocial stress may contribute to poorer self-care, tobacco or alcohol use, altered salivary flow, and inflammatory responses. Behavioral exposures, including tobacco smoke exposure, have also been described in the caries literature [6]. In parallel, cardiometabolic conditions such as dysglycemia, obesity-related markers, and elevated blood pressure may share inflammatory or behavioral pathways with oral diseases. Therefore, oral health events in workers should not be understood as strictly occupational diseases, but as conditions potentially patterned by occupational organization, health behaviors, access to care, and general health status.

Despite the magnitude of the problem and the relevance of the work environment, there is a specific gap in evidence on the incidence of clinically detected caries and gingivitis in occupational cohorts with repeated assessments. Most global information is based on prevalence, whereas longitudinal studies with repeated measurements in adult workers remain scarce and heterogeneous. This limits the identification of worker subgroups with higher observed event rates and makes it difficult to plan risk-proportionate surveillance, preventive education, and referral pathways in everyday occupational health practice [2,3,4,5,7].

Given this gap, the present study aimed to estimate the incidence of dental caries and gingivitis, as well as a combined oral health outcome, in a cohort of workers undergoing periodic occupational health evaluations, and to describe demographic, clinical, behavioral, and occupational differences in the occurrence of these events. Estimating incidence, rather than only baseline prevalence, is relevant for occupational medicine because it identifies new events arising during follow-up, supports the planning of risk-proportionate surveillance intervals, and helps prioritize preventive education or referral strategies for worker subgroups with higher observed event rates.

## 2. Methodology

### 2.1. Type and Design of Research

This was a retrospective descriptive cohort study based on a secondary analysis of clinical records from an occupational health clinic between 2014 and 2021. The design and reporting of results followed the STROBE recommendations for observational studies [8] (Supplementary Material Table S1). The primary objective was to estimate incident oral health events and describe crude differences in their occurrence across demographic, clinical, behavioral, and occupational characteristics. The study was not designed to estimate causal effects; therefore, the comparative estimates should be interpreted as descriptive crude contrasts rather than adjusted or causal associations [9,10].

### 2.2. Population and Sample

The target population consisted of all workers affiliated with companies that require occupational health assessments. The accessible population corresponded to workers who attended their assessments at the occupational polyclinic, carried out annually or biennially in accordance with company requirements and current labor regulations. The study population consisted of those who met the selection criteria; consecutive non-probabilistic sampling included all workers evaluated during the study period who met the eligibility conditions. The database classified workers according to broad occupational activity categories but did not contain standardized economic sector or industry codes; therefore, industry-specific representativeness could not be formally assessed.

### 2.3. Selection Criteria

Workers with at least two separate evaluations, with a minimum interval of one year and with a complete record of the variables essential for the analysis, were included. Given the interest in estimating the incidence, for each outcome, those who already presented the event at the baseline evaluation, either the presence of caries or gingivitis, were excluded from the risk. This strategy allowed us to construct, for each outcome, a subcohort free of the event at the beginning.

### 2.4. Follow-Up and Definition of Events

Follow-up of each participant began at the first recorded assessment (baseline) and ended at the last available assessment or at the time of the first clinical detection of the event of interest by the occupational dentist. Because workers had variable follow-up times depending on company requirements and the timing of repeated occupational evaluations, incidence was estimated using accumulated person-time. The event date corresponded to the date of clinical detection during an occupational dental evaluation; therefore, the time-to-event analyses should be interpreted as time to clinical detection rather than exact time to biological onset. If the event occurred, the participant ceased to contribute time at risk for that specific outcome; if no event was detected, the participant was censored at the last available dental evaluation.

### 2.5. Statistical Accuracy

Since this is a retrospective analysis with consecutive sampling, no a priori sample size calculation was performed; all available records that met the eligibility criteria were included. The precision of the incidence estimates was assessed using 95% confidence intervals obtained by exact methods based on the Poisson distribution, considering the number of events and the cumulative follow-up time. From a methodological point of view, the relative precision (95% CI half-width divided by the estimated rate) of an incidence rate depends mainly on the event count (E) and is approximated by 1.96/√E [11].

The cohorts analyzed included 11,888 participants for caries, 11,984 for gingivitis, and 11,851 for the combined outcome. With the events observed in each analysis (caries: E = 470; gingivitis: E = 149; combined: E = 542), the expected relative precision is approximately 9.0% for caries (1.96/√470), 16.1% for gingivitis (1.96/√149), and 8.4% for the combined outcome (1.96/√542). These relative margins of error are consistent with event counts in the hundreds and are considered adequate for the descriptive purposes of the study. The exact values of the rates and confidence intervals, along with the corresponding corrected person-time, are presented in Section 3 [11].

### 2.6. Variables and Measurement

The main outcomes were caries and gingivitis. Caries detection was based on direct visual–tactile clinical examination by occupational dentists, with the presence of cavitated lesions in enamel or dentin requiring restorative intervention according to clinical criteria being considered positive. Gingivitis was diagnosed by visual inspection of the gingival tissue during the occupational dental examination and was recorded as present when the dentist identified clinical signs of gingival inflammation, including discoloration, edema, spontaneous bleeding or bleeding on gentle probing, and the presence of bacterial plaque or dental calculus. These criteria correspond to the usual practice of dental screening in the context of occupational health in the country, where the objective is to identify conditions that require timely referral or treatment, rather than staging severity using complex indices.

Early non-cavitated enamel lesions, caries experience indices such as DMFT/DMFS, lesion severity staging, root caries as a separate outcome, and radiographic detection were not systematically recorded in the occupational screening database. Therefore, the outcomes should be interpreted as clinically detected screening events requiring referral or treatment, rather than as the full epidemiological burden of dental caries experience or gingival inflammation. This distinction is important because deep or cavitated carious lesions requiring treatment represent a more clinically selected subset of the caries spectrum [12].

The reference characteristics included multiple dimensions assessed at baseline. Demographic variables included sex, categorized as male or female, and age recorded in years at the time of the first evaluation. Clinical parameters were measured using standardized protocols at the initial assessment. Systolic and diastolic blood pressure were recorded in millimeters of mercury (mmHg) as continuous variables. Waist circumference was measured in centimeters at the midpoint between the lower costal margin and the iliac crest, according to standardized anthropometric techniques. Biochemical markers included fasting glucose, total cholesterol, and triglycerides, all quantified in milligrams per deciliter (mg/dL) using venous blood samples collected after an 8–12 h fast, and were treated as continuous variables in the analyses. Body mass index (BMI) was calculated by dividing weight in kilograms by the square of height in meters (kg/m^2^).

Behavioral characteristics were documented using a standardized occupational history questionnaire. Smoking was assessed by reporting cigarette consumption in the last 30 days prior to the evaluation, categorized as current smoker (any reported consumption) or nonsmoker. Alcohol consumption was categorized as present or absent according to the occupational questionnaire report. Consumption was considered present when the worker reported drinking alcoholic beverages at least once a month during the past year, or when there was an episode of excessive consumption (defined as five or more standard drinks on a single occasion for men, or four or more for women) in the past 30 days.

Occupational variables characterized the work environment and type of work. The type of occupation was classified into four mutually exclusive categories according to the main work activity reported: (1) Office/administrative, which includes desk workers and administrative tasks; (2) Manual/physical, which groups occupations that require physical effort and predominantly manual work; (3) Customer service/sales or social services, which encompasses activities involving direct customer service and sales; and (4) Healthcare professional, which includes doctors, nurses, and other healthcare professionals. Years of occupational exposure were recorded as the time elapsed from the start of the worker’s current job to the date of the baseline assessment and were categorized into four groups: less than 1 year, 1 to <5 years, 5 to <10 years, and ≥10 years, allowing description of incidence patterns by job tenure. Night work was defined as work performed predominantly between 8:00 p.m. and 7:00 a.m., determined by the question “Do you currently work mainly at night or during the day?”, and was categorized dichotomously as present or absent, regardless of occasional additional day shifts.

All these variables were used as descriptive characteristics of the cohort and as markers for crude time-to-event contrasts with the outcomes of interest, without attempting to establish causal relationships.

### 2.7. Procedures

The evaluations followed the routine of the occupational clinic. After a standardized questionnaire on sociodemographic, occupational, and health history, the dental examination was performed by licensed dentists with experience in occupational health, using artificial light, a dental mirror, and an explorer. The evaluation included a systematic inspection of all accessible tooth surfaces to detect carious lesions and an examination of the gingival condition in all quadrants. Diagnoses were recorded in the institutional electronic medical record using dichotomous categories of presence or absence, reflecting the pragmatic approach of occupational screening geared toward clinical referral decisions. Because this was a retrospective analysis of routine clinical records, no formal inter-examiner calibration exercise or agreement statistics, such as kappa coefficients, were available for the study period.

Anthropometric measurements (weight, height, and waist circumference) were taken by technical staff following standardized protocols. Biochemical parameters (glucose and lipid profile) were obtained by venous blood sampling after an 8–12 h fast and processed in the clinic laboratory according to established protocols. Finally, the occupational medical evaluation documented lifestyle habits and job characteristics, including exposure to night shifts and time at work.

### 2.8. Statistical Analysis

Processing was performed in R. (version 4.3.2; R Foundation for Statistical Computing, Vienna, Austria). Baseline characteristics were described using frequencies and percentages for categorical variables and means with standard deviations for continuous variables. Follow-up time was calculated from the baseline dental evaluation to the date of first event detection or the last available dental evaluation. In the analytical dataset, follow-up was converted to person-years using the date-based scale before estimating incidence rates. Incidence rates per 1000 person-years were estimated with 95% CIs based on the Poisson distribution.

Crude HRs and 95% CIs were calculated using univariate Cox proportional hazards models, with one characteristic entered at a time. These models were used as descriptive time-to-event contrasts and not as causal models. Therefore, no multivariable adjustment strategy was implemented. For continuous variables, HRs were expressed in increments of 5 units, and continuous variables were categorized into quartiles only for descriptive presentation of incidence patterns.

To improve interpretation of the magnitude of observed contrasts, HRs were interpreted together with absolute incidence rates, rate differences per 1000 person-years, and supplementary age- and sex-standardized rates. Because the outcomes were time-to-event screening diagnoses, standardized mean-difference measures such as Cohen’s d or Hedges’ g were not considered appropriate effect-size metrics for the primary analyses, despite their value for continuous outcomes in dental research [13].

As supplementary descriptive analyses, age- and sex-standardized incidence rates were estimated by direct standardization using the outcome-specific analytical cohort as the standard population. Standardization strata were defined by sex and age group (18–29, 30–39, 40–49, and ≥50 years). Standardized rate differences and ratios were used as descriptive measures of magnitude and were not interpreted as causal or inferential estimates.

### 2.9. Ethical Aspects

The protocol was approved by the Research Ethics Committee of the Toribio Rodríguez de Mendoza National University. All data were analyzed anonymously, without personal or company identifiers. The study was conducted in accordance with the Declaration of Helsinki and good occupational health research practices. For transparency and reproducibility purposes, the anonymized database is available in open access in the repository indicated by the authors [14].

## 3. Results

Of the 79,660 workers evaluated at the occupational clinic during the study period, 12,049 met the eligibility criteria by having at least two dental evaluations separated by a minimum of one year and complete records of essential variables. The remaining 67,611 workers were excluded because they did not have valid follow-up. From this cohort with follow-up, three outcome-specific subcohorts were formed according to baseline status: 11,888 workers free of caries at baseline, 11,984 workers free of gingivitis at baseline, and 11,851 workers free of either condition at baseline (see Figure 1). The median follow-up time was 2.16 years (IQR 1.85–3.37). By occupation, the median follow-up was 2.22 years (IQR 1.89–3.91) for office/administrative workers, 2.12 years (IQR 1.74–3.02) for manual/physical workers, 2.50 years (IQR 2.06–3.90) for customer service/social service workers, and 2.09 years (IQR 1.87–2.90) for healthcare professionals. The median number of dental evaluations per worker was 2 (IQR 2–3); 70.4% of workers had two evaluations and 29.6% had three or more evaluations.

The cohort consisted of 12,049 workers, predominantly male (84.87%), with an average age of 37.2 years. More than half performed manual or physical occupations (54.64%), followed by office/administrative workers (36.16%), while health professionals represented only 2.36% of the sample. Most did not work night shifts (93.41%) and had less than one year of occupational exposure at the time of the initial assessment (57.65%). Regarding habits, 40.96% reported active smoking and approximately half consumed alcohol. In terms of clinical parameters, the baseline means were: systolic blood pressure 111.3 mmHg, diastolic blood pressure 71.9 mmHg, waist circumference 91.2 cm, body mass index (BMI) 27.2 kg/m^2^, glucose 92.4 mg/dL, total cholesterol 194.8 mg/dL, and triglycerides 143.6 mg/dL. At baseline, the prevalence of caries was 1.34%, gingivitis 0.54%, and the combined outcome 1.64% (Table 1).

The overall incidence rate of caries was 14.86 cases per 1000 person-years (95% CI 13.55–16.27), with 470 events observed during 31,627.3 person-years of follow-up. Male workers had higher observed incidence than female workers (15.96 vs. 8.73 per 1000 person-years; crude HR 1.81, 95% CI 1.32–2.48). No clear crude differences were observed for smoking, alcohol consumption, or night work. By occupation type, manual/physical workers had higher observed incidence than office/administrative workers (18.87 vs. 11.62 per 1000 person-years; crude HR 1.93, 95% CI 1.58–2.36), and healthcare professionals also showed higher observed incidence, although with fewer events. Customer service/social service workers had very few events and a low observed rate; therefore, this estimate should be interpreted cautiously. A graded pattern was observed with years of occupational exposure: compared with workers with <1 year, crude HRs were 1.75 (95% CI 1.34–2.29) for 1 to <5 years, 3.21 (95% CI 2.42–4.25) for 5 to <10 years, and 3.61 (95% CI 2.87–4.53) for ≥10 years. Regarding continuous variables, age, baseline glucose, and total cholesterol showed positive crude contrasts with caries. Waist circumference, systolic blood pressure, diastolic blood pressure, and triglycerides did not show clear crude contrasts when analyzed as continuous variables (Table 2).

The overall incidence rate of gingivitis was 4.67 cases per 1000 person-years (95% CI 3.95–5.49), with 149 events observed during 31,892.7 person-years of follow-up. Male workers had higher observed incidence than female workers (4.95 vs. 3.10 per 1000 person-years), although the crude HR was imprecise (HR 1.55, 95% CI 0.91–2.65). No clear crude differences were observed for smoking, alcohol consumption, or night work. By occupation type, the crude difference between manual/physical and office/administrative workers was modest for gingivitis (HR 1.26, 95% CI 0.90–1.76). Customer service/social service workers and healthcare professionals had few events, and their estimates should be interpreted cautiously. A graded pattern was observed with years of occupational exposure, with crude HRs of 2.28 (95% CI 1.49–3.47) for 1 to <5 years, 2.10 (95% CI 1.19–3.73) for 5 to <10 years, and 3.11 (95% CI 2.05–4.72) for ≥10 years compared with <1 year. Regarding continuous variables, systolic blood pressure showed a positive crude contrast with gingivitis, whereas age, waist circumference, glucose, triglycerides, and total cholesterol did not show clear crude contrasts (Table 3).

The incidence rate of the combined outcome was 17.20 cases per 1000 person-years (95% CI 15.78–18.71), with 542 events observed during 31,513.8 person-years of follow-up. Male workers had higher observed incidence than female workers (18.38 vs. 10.61 per 1000 person-years; crude HR 1.72, 95% CI 1.29–2.29). No clear crude differences were observed for smoking, alcohol consumption, or night work. By occupation type, manual/physical workers had higher observed incidence than office/administrative workers (21.33 vs. 14.31 per 1000 person-years; crude HR 1.77, 95% CI 1.47–2.12), and healthcare professionals also showed higher observed incidence, although based on few events. A graded pattern was maintained across years of occupational exposure. Regarding continuous variables, age, baseline glucose, total cholesterol, systolic blood pressure, and triglycerides showed positive crude contrasts with the combined outcome, whereas waist circumference and diastolic blood pressure did not show clear crude contrasts (Table 4).

In supplementary age- and sex-standardized analyses, the occupational contrast was clearer for caries than for gingivitis. Standardized caries rates were 11.70 per 1000 person-years among office/administrative workers, 18.71 among manual/physical workers, and 21.80 among healthcare professionals. In contrast, standardized gingivitis rates were similar between office/administrative and manual/physical workers (4.71 vs. 4.84 per 1000 person-years). Longer occupational exposure showed a clearer graded pattern for both outcomes, with standardized rates increasing from 9.77 to 27.67 per 1000 person-years for caries and from 3.05 to 7.46 per 1000 person-years for gingivitis between <1 year and ≥10 years of occupational exposure (Supplementary Table S2).

## 4. Discussion

### 4.1. Main Findings

This study provides longitudinal descriptive evidence on clinically detected caries and gingivitis events among workers undergoing periodic occupational health evaluations. After correcting the person-time denominator to person-years, the incidence rates were 14.86 per 1000 person-years for caries, 4.67 per 1000 person-years for gingivitis, and 17.20 per 1000 person-years for the combined outcome. Incident oral health events were not uniformly distributed across worker characteristics. Higher observed incidence was found among men, manual/physical workers, workers with longer job tenure, and workers with selected cardiometabolic markers. However, because the analyses were crude and no causal adjustment strategy was implemented, these findings should be interpreted as descriptive patterns of occurrence rather than independent risk factors or causal effects.

### 4.2. Contrast with the Literature: Incidence in Occupational Settings

The literature on the incidence of oral diseases in the working population is notably scarce, with cross-sectional prevalence studies predominating. A Swedish registry-based cohort study reported an incidence of periodontitis of 9.8% during 12 years of follow-up in young adults, with smoking and periodontal pocket depth in adolescence as significant risk factors [15]. Although that study addressed advanced periodontitis rather than incipient gingivitis, its findings support the importance of longitudinal follow-up to capture the natural history of periodontal diseases. In a 26-year longitudinal cohort of Norwegian males, persistent gingival inflammation was associated with greater attachment loss and lower tooth survival, supporting the notion that persistent gingivitis is a periodontal risk factor [16].

Cross-sectional studies in workers have documented associations between occupational parameters and oral health. Zaitsu et al., in a study of 1078 Japanese industrial workers, found that not receiving instruction in tooth brushing (OR 1.73) and not having annual dental examinations (OR 1.64) were associated with caries, while working in small companies was related to severe periodontal disease (OR 15.56) [17]. These findings are consistent with the relevance of workplace organization and preventive access, although our study should be interpreted as estimating longitudinal incidence and crude descriptive contrasts rather than causal occupational effects. A systematic review on work stress and oral health identified significant associations between occupational stress and poorer periodontal status in eight of nine studies analyzed, while evidence on caries was scarce [18]. In construction workers in India, high caries prevalence has also been described, suggesting that occupational conditions may shape oral health through multiple behavioral and environmental pathways [19].

The crude positive contrast between baseline glucose and incident caries observed in our study is compatible with biological and epidemiological evidence linking dysglycemia and oral health. Cross-sectional studies have documented that patients with type 2 diabetes have higher levels of caries than healthy individuals, associating this with hyperglycemia, reduced salivary flow, and increased glucose in saliva, which may favor cariogenic bacterial metabolism [20,21]. Experimental evidence in diabetic animal models has also suggested that prolonged hyperglycemia may induce dental caries through pathways distinct from periodontal disease [22]. Nevertheless, our data did not include HbA1c, salivary biomarkers, diet, or oral hygiene practices; therefore, baseline glucose should be interpreted as a descriptive cardiometabolic marker of higher observed caries incidence rather than as an independent causal factor.

The crude contrast between elevated systolic blood pressure and incident gingivitis is consistent with the literature linking periodontal inflammation and blood pressure. Proposed mechanisms include low-grade systemic inflammation, endothelial dysfunction, immune response to periodontal pathogens, and alterations in nitric oxide production [23,24,25,26]. However, causal directionality cannot be established from the present study. Blood pressure and gingival inflammation may share behavioral, inflammatory, or healthcare-access pathways, and unmeasured confounding remains likely.

The graded pattern observed between years of occupational exposure and both oral outcomes was one of the most notable descriptive findings of the study. Compared with workers with <1 year of exposure, those with ≥10 years had crude HRs of 3.61 for caries and 3.11 for gingivitis. In absolute terms, supplementary standardized analyses showed 17.90 additional caries events and 4.41 additional gingivitis events per 1000 person-years among workers with ≥10 years compared with those with <1 year of exposure. This pattern may reflect accumulation of occupational and behavioral constraints over working life, but it may also be influenced by age structure, retention in employment, healthy-worker effects, company-level policies, visit frequency, or unmeasured socioeconomic and behavioral variables. Therefore, this finding should be interpreted as a graded descriptive pattern, not as a demonstrated causal exposure effect.

The estimates for customer service/social service workers and healthcare professionals should be interpreted cautiously because they were based on few events. Although the observed incidence was low among customer service/social service workers and relatively higher among healthcare professionals, these contrasts may be unstable and susceptible to sparse-data bias. The study did not measure appearance-related self-care, occupational stress, dietary patterns, or oral hygiene practices; therefore, explanations for these subgroup patterns should be regarded as hypotheses for future research rather than conclusions supported by the present data.

### 4.3. Implications for Public and Occupational Health

Before discussing the practical implications of these results, it is essential to contextualize their scope. The study population was predominantly male and included a high proportion of manual/physical workers, reflecting the composition of the source occupational clinic rather than a probability sample of the entire workforce. Because the dataset included broad occupational categories but no standardized industry or economic-sector codes, industry-specific representativeness could not be formally assessed. The findings are therefore most directly applicable to workers undergoing periodic occupational health evaluations in the source setting.

The results may inform the design of occupational oral health programs by identifying worker subgroups with higher observed incidence of clinically detected oral health events. Manual/physical workers and workers with longer job tenure may warrant closer preventive attention, education, or referral pathways. However, the present study did not evaluate the effectiveness, feasibility, or cost-effectiveness of differentiated surveillance intervals, and its estimates should not be interpreted as evidence that intensified surveillance would necessarily improve outcomes.

The observed crude contrasts involving metabolic and cardiovascular markers support the conceptual integration of oral health with general occupational health surveillance. Occupational programs could consider coordinated referral or education when dental findings coexist with cardiometabolic markers. Nevertheless, this interpretation should remain descriptive because the study did not measure inflammatory biomarkers, diet, salivary function, medication use, or external dental-care utilization.

The occupational setting offers a useful platform for public health interventions because it allows regular contact with adult workers who may not otherwise receive preventive services. Although productivity, absenteeism, presenteeism, and treatment costs were not measured in this study, they provide an important occupational health rationale for monitoring oral health in workers. Whether intensified surveillance or workplace preventive programs reduce dental emergencies, productivity loss, or costs requires prospective intervention and economic evaluation studies.

Occupational health policies could benefit from future evidence on optimal assessment intervals differentiated by observed event rates rather than uniform schemes. A stratified surveillance model may optimize resources by intensifying follow-up for those who need it most, but such a model would require prospective validation, standardized oral health indices, richer behavioral and occupational exposure data, and cost-effectiveness evaluation before implementation.

### 4.4. Limitations

Several limitations should be considered. First, caries and gingivitis were identified through routine occupational screening and recorded dichotomously; standardized indices such as ICDAS, DMFT/DMFS, or quantified periodontal indices were not available. Consequently, the low baseline prevalence and incidence estimates should be interpreted as clinically detected occupational screening events rather than the full epidemiological burden of caries experience, non-cavitated enamel lesions, radiographic lesions, periodontal severity, or gingival inflammation. Second, no formal inter-examiner calibration statistics were available, which may have introduced diagnostic variability, particularly for gingivitis. Third, events were dated at clinical detection during annual or biennial evaluations, so the true biological onset may have occurred between visits, introducing interval censoring. Therefore, the time-to-event analyses should be interpreted as time to clinical detection rather than exact time to disease onset.

Fourth, only workers with valid repeated evaluations and complete essential records were included. Workers retained in periodic surveillance may belong to more stable companies, have more formal employment conditions, different occupational risks, or better access to healthcare than workers with only one evaluation or incomplete follow-up. This selection process may limit generalizability and could bias incidence estimates if follow-up availability was related to oral health status or occupational conditions. Finally, relevant behavioral and contextual variables, including toothbrushing frequency, diet, dental visits outside the occupational setting, company-level policies, company identifiers for clustered analyses, and standardized industry codes, were unavailable; therefore, residual confounding, underestimated standard errors due to unmeasured clustering, and limited generalizability should be expected.

## 5. Conclusions

This retrospective descriptive cohort study documented incident clinically detected dental caries and gingivitis among workers undergoing periodic occupational health evaluations. Higher observed incidence was particularly evident for caries among manual/physical workers and for both oral outcomes among workers with longer occupational exposure. Occupational differences in gingivitis were smaller after age and sex standardization and should be interpreted cautiously. Selected cardiometabolic markers also showed crude descriptive contrasts with oral health outcomes, but these estimates should not be interpreted as causal effects.

These findings may help occupational health services identify worker subgroups that warrant closer preventive attention, oral health education, or referral pathways. However, the effectiveness, feasibility, and cost-effectiveness of differentiated surveillance strategies should be evaluated in future prospective and intervention studies using standardized oral health indices, behavioral measures, specific occupational exposure data, company-level information, and causal modeling guided by explicit assumptions.

## Figures and Tables

**Figure 1:**
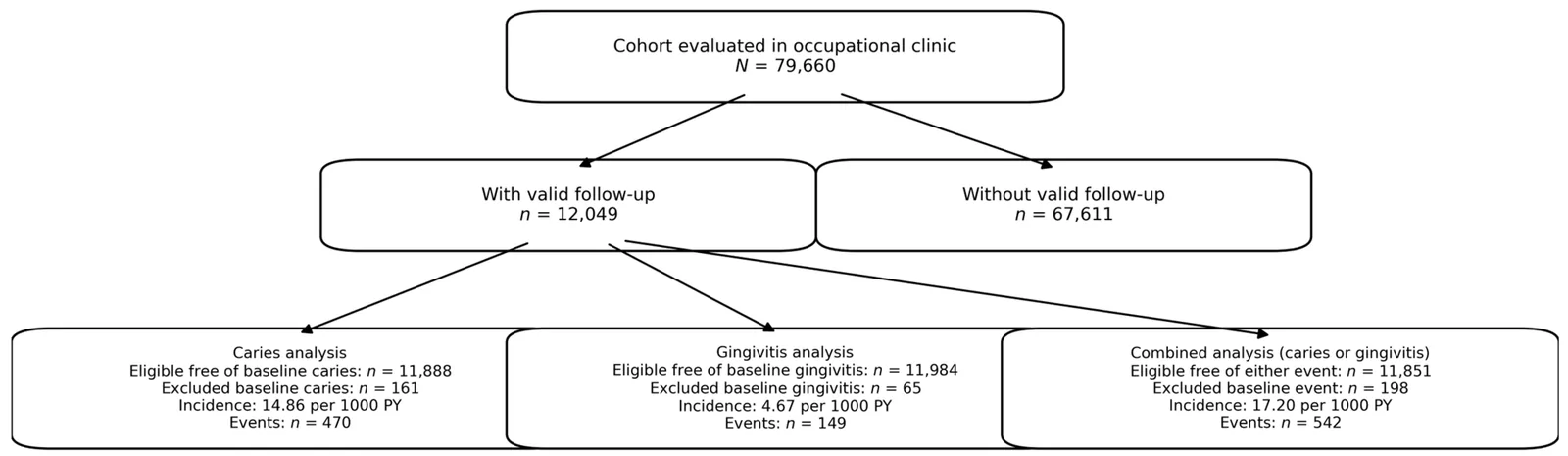
Flow chart of participant selection with corrected incidence rates per 1000 person-years.

**Table 1:** Characteristics of study participants.

Characteristics	*n* = 12,049
**Sex**	
Female	1823 (15.13%)
Male	10,226 (84.87%)
**Age (years)**	37.19 (11.56)
**Type of occupation**	
Office	4357 (36.16%)
Manual/Physical	6584 (54.64%)
Customer service/Social services	824 (6.83%)
Healthcare professional	284 (2.36%)
**Night work**	
No	11,255 (93.41%)
Yes	794 (6.59%)
**Years of exposure**	
Less than 1 year	6946 (57.65%)
1 to <5 years	2487 (20.64%)
5 to <10 years	1105 (9.17%)
≥10 years	1511 (12.54%)
**Smoker**	
No	7114 (59.04%)
Yes	4935 (40.96%)
**Alcohol consumption**	
No	6031 (50.05%)
Yes	6018 (49.95%)
**Systolic blood pressure (mmHg)**	111.27 (12.26)
**Diastolic blood pressure (mmHg)**	71.94 (13.35)
**Waist circumference (cm)**	91.15 (10.41)
Body mass index (BMI) (kg/m^2^)	27.15 (3.99)
**Glucose (mg/dL)**	92.44 (20.80)
**Total cholesterol (mg/dL)**	194.83 (36.83)
**Triglycerides (mg/dL)**	143.63 (80.93)
**Caries (baseline)**	
No	11,888 (98.66%)
Yes	161 (1.34%)
**Gingivitis (baseline)**	
No	11,984 (99.46%)
Yes	65 (0.54%)
**Caries or gingivitis (baseline)**	
No	11,851 (98.36%)
Yes	198 (1.64%)

*n* (%); Mean (SD). BMI = body mass index.

**Table 2:** Incidence of caries per 1000 person-years according to demographic, occupational, and clinical characteristics.

Characteristics	Incidence Rate (95% CI)	Events	PY	Crude HR (95% CI)
Overall incidence	14.86 (13.55–16.27)	470	31,627.3	
**Sex**				
Female	8.73 (6.29–11.80)	42	4810.9	1.00 (Ref.)
Male	15.96 (14.48–17.55)	428	26,816.4	1.81 (1.32–2.48)
**Smoker**				
No	14.27 (12.60–16.09)	265	18,574.4	1.00 (Ref.)
Yes	15.71 (13.63–18.01)	205	13,053.0	1.08 (0.90–1.30)
**Alcohol consumption**				
No	14.52 (12.70–16.52)	230	15,845.3	1.00 (Ref.)
Yes	15.21 (13.34–17.26)	240	15,781.9	1.05 (0.88–1.26)
**Night work**				
No	14.83 (13.48–16.29)	438	29,528.8	1.00 (Ref.)
Yes	15.25 (10.43–21.53)	32	2098.5	0.94 (0.65–1.34)
**Type of occupation**				
Office	11.62 (9.78–13.70)	141	12,134.8	1.00 (Ref.)
Manual/Physical	18.87 (16.83–21.09)	311	16,480.2	1.93 (1.58–2.36)
Customer service/Social services	1.30 (0.27–3.81)	3	2301.4	0.14 (0.04–0.43)
Healthcare professional	21.10 (11.81–34.80)	15	710.9	2.18 (1.28–3.71)
**Years of exposure**				
<1 year	10.16 (8.75–11.73)	186	18,302.9	1.00 (Ref.)
1 to <5 years	12.67 (10.01–15.81)	78	6156.4	1.75 (1.34–2.29)
5 to <10 years	24.16 (18.80–30.57)	69	2856.4	3.21 (2.42–4.25)
≥10 years	31.78 (26.68–37.56)	137	4311.5	3.61 (2.87–4.53)
**Age in years**				
Per 5-year increase				1.07 (1.03–1.11)
**Incidence by quartiles**				
Q1: 18–28	11.37 (9.18–13.91)	94	8270.6	—
Q2: >28–36	14.47 (11.95–17.37)	115	7947.8	—
Q3: >36–45	14.61 (12.00–17.60)	110	7531.5	—
Q4: >45–76	19.17 (16.23–22.48)	151	7877.3	—
**Abdominal waist**				
Per 5 cm increase				0.99 (0.94–1.04)
**Incidence by quartiles**				
Q1: 53–84	18.49 (15.39–22.03)	125	6761.0	—
Q2: >84–91	17.85 (14.75–21.41)	116	6497.5	—
Q3: >91–98	14.62 (11.76–17.97)	90	6155.7	—
Q4: >98–147	14.51 (11.54–18.01)	82	5650.4	—
**Systolic blood** **pressure**				
Per 5 mmHg increase				1.02 (0.99–1.06)
**Incidence by quartiles**				
Q1: 83–103	12.85 (11.01–14.91)	173	13,464.6	—
Q2: >103–113	16.33 (13.73–19.28)	139	8511.8	—
Q3: >113–123	16.49 (13.67–19.72)	120	7277.5	—
Q4: >123–202	15.89 (11.19–21.90)	37	2329.0	—
**Diastolic blood pressure**				
Per 5 mmHg increase				1.01 (0.97–1.05)
**Incidence by quartiles**				
Q1: 53–63	14.63 (12.60–16.90)	185	12,642.7	—
Q2: >63–73	12.98 (10.87–15.37)	134	10,326.5	—
Q3: >73–83	18.79 (15.75–22.24)	135	7184.7	—
Q4: >83–123	10.61 (5.94–17.51)	15	1413.3	—
**Glucose**				
Per 5 mg/dL increase				1.04 (1.02–1.06)
**Incidence by quartiles**				
Q1: 56–83.1	7.36 (5.54–9.58)	55	7476.6	—
Q2: >83.1–91	9.82 (7.59–12.49)	66	6721.3	—
Q3: >91–98	11.35 (9.07–14.04)	85	7487.8	—
Q4: >98–377.7	16.03 (12.69–19.98)	79	4929.0	—
**Triglycerides**				
Per 5 mg/dL increase				1.01 (1.00–1.01)
**Incidence by quartiles**				
Q1: 27–89	11.24 (8.31–14.86)	49	4360.4	—
Q2: >89–124	11.47 (8.48–15.16)	49	4272.4	—
Q3: >124–178	14.63 (11.21–18.75)	62	4239.3	—
Q4: >178–957	15.14 (11.64–19.37)	63	4160.8	—
**Total cholesterol**				
Per 5 mg/dL increase				1.02 (1.01–1.04)
**Incidence by quartiles**				
Q1: 79–167	10.29 (7.54–13.73)	46	4469.2	—
Q2: >167–191	12.01 (8.94–15.79)	51	4246.8	—
Q3: >191–219.45	15.59 (12.04–19.88)	65	4168.2	—
Q4: >219.45–456.62	14.65 (11.21–18.82)	61	4164.0	—

Note: PY = person-years. Incidence rates are per 1000 PY. HR = hazard ratio; CI = confidence interval; Ref. = reference category.

**Table 3:** Incidence of gingivitis per 1000 person-years according to demographic, occupational, and clinical characteristics.

Characteristics	Incidence Rate (95% CI)	Events	PY	Crude HR (95% CI)
Overall incidence	4.67 (3.95–5.49)	149	31,892.7	
**Sex**				
Female	3.10 (1.74–5.12)	15	4835.2	1.00 (Ref.)
Male	4.95 (4.15–5.87)	134	27,057.6	1.55 (0.91–2.65)
**Smoker**				
No	4.75 (3.82–5.85)	89	18,728.0	1.00 (Ref.)
Yes	4.56 (3.48–5.87)	60	13,164.7	0.94 (0.68–1.31)
**Alcohol consumption**				
No	4.69 (3.69–5.88)	75	15,975.5	1.00 (Ref.)
Yes	4.65 (3.65–5.84)	74	15,917.2	0.99 (0.72–1.36)
**Night work**				
No	4.74 (3.99–5.58)	141	29,774.6	1.00 (Ref.)
Yes	3.78 (1.63–7.44)	8	2,118.1	0.74 (0.36–1.52)
**Type of occupation**				
Office	4.74 (3.60–6.12)	58	12,247.7	1.00 (Ref.)
Manual/Physical	5.12 (4.09–6.33)	85	16,614.5	1.26 (0.90–1.76)
Customer service/Social services	0.43 (0.01–2.40)	1	2320.3	0.11 (0.02–0.79)
Healthcare professional	7.04 (2.29–16.43)	5	710.2	1.75 (0.70–4.36)
**Years of exposure**				
<1 year	3.30 (2.53–4.24)	61	18,465.2	1.00 (Ref.)
1 to <5 years	5.63 (3.92–7.83)	35	6216.4	2.28 (1.49–3.47)
5 to <10 years	5.24 (2.93–8.64)	15	2864.6	2.10 (1.19–3.73)
≥10 years	8.74 (6.19–12.00)	38	4346.4	3.11 (2.05–4.72)
**Age in years**				
Per 5-year increase				1.04 (0.97–1.11)
**Incidence by quartiles**				
Q1: 18–28	3.61 (2.44–5.16)	30	8303.7	—
Q2: >28–36	4.98 (3.56–6.78)	40	8031.7	—
Q3: >36–45	4.73 (3.31–6.54)	36	7615.2	—
Q4: >45–76	5.41 (3.92–7.29)	43	7942.1	—
**Abdominal waist**				
Per 5 cm increase				0.98 (0.89–1.09)
**Incidence by quartiles**				
Q1: 53–84	4.40 (2.97–6.28)	30	6816.2	—
Q2: >84–91	4.12 (2.72–6.00)	27	6547.4	—
Q3: >91–98	2.43 (1.36–4.01)	15	6175.4	—
Q4: >98–147	4.03 (2.56–6.05)	23	5705.0	—
**Systolic blood pressure**				
Per 5 mmHg increase				1.07 (1.01–1.13)
**Incidence by quartiles**				
Q1: 83–103	3.32 (2.42–4.44)	45	13,561.1	—
Q2: >103–113	5.00 (3.62–6.74)	43	8592.5	—
Q3: >113–123	7.35 (5.52–9.60)	54	7342.8	—
Q4: >123–202	2.98 (1.20–6.13)	7	2352.0	—
**Diastolic blood pressure**				
Per 5 mmHg increase				1.03 (1.00–1.06)
**Incidence by quartiles**				
Q1: 53–63	3.15 (2.25–4.29)	40	12,710.7	—
Q2: >63–73	5.37 (4.06–6.98)	56	10,425.5	—
Q3: >73–83	6.75 (4.99–8.92)	49	7259.0	—
Q4: >83–123	2.78 (0.76–7.12)	4	1437.5	—
**Glucose**				
Per 5 mg/dL increase				1.00 (0.96–1.05)
**Incidence by quartiles**				
Q1: 56–83.1	3.57 (2.35–5.19)	27	7568.3	—
Q2: >83.1–91	5.62 (3.98–7.72)	38	6756.4	—
Q3: >91–98	4.38 (3.01–6.15)	33	7535.2	—
Q4: >98–377.7	2.62 (1.39–4.48)	13	4965.9	—
**Triglycerides**				
Per 5 mg/dL increase				1.01 (1.00–1.02)
**Incidence by quartiles**				
Q1: 27–89	3.89 (2.27–6.23)	17	4370.8	—
Q2: >89–124	3.46 (1.94–5.71)	15	4333.0	—
Q3: >124–178	3.98 (2.32–6.37)	17	4273.8	—
Q4: >178–957	4.52 (2.72–7.05)	19	4205.8	—
**Total cholesterol**				
Per 5 mg/dL increase				1.01 (0.98–1.05)
**Incidence by quartiles**				
Q1: 79–167	3.33 (1.86–5.49)	15	4506.2	—
Q2: >167–191	5.38 (3.41–8.07)	23	4276.4	—
Q3: >191–219.45	3.72 (2.21–5.88)	18	4834.2	—
Q4: >219.45–456.20	3.36 (1.73–5.86)	12	3575.4	—

Note: PY = person–years. Incidence rates are per 1000 PY. HR = hazard ratio; CI = confidence interval; Ref. = reference category.

**Table 4:** Incidence of the combined outcome per 1,000 person-years according to demographic, occupational, and clinical characteristics.

Characteristics	Incidence Rate (95% CI)	Events	PY	Crude HR (95% CI)
Overall incidence	17.20 (15.78–18.71)	542	31,513.8	
**Sex**				
Female	10.61 (7.90–13.95)	51	4806.2	1.00 (Ref.)
Male	18.38 (16.79–20.08)	491	26,707.7	1.72 (1.29–2.29)
**Smoker**				
No	16.59 (14.78–18.55)	307	18,507.0	1.00 (Ref.)
Yes	18.07 (15.83–20.53)	235	13,006.8	1.07 (0.90–1.27)
**Alcohol consumption**				
No	16.90 (14.93–19.05)	267	15,801.2	1.00 (Ref.)
Yes	17.50 (15.49–19.70)	275	15,712.6	1.04 (0.88–1.23)
**Night work**				
No	17.24 (15.77–18.80)	508	29,474.7	1.00 (Ref.)
Yes	16.67 (11.55–23.30)	34	2039.1	0.89 (0.63–1.27)
**Type of occupation**				
Office	14.31 (12.25–16.60)	173	12,092.5	1.00 (Ref.)
Manual/Physical	21.33 (19.15–23.68)	350	16,411.7	1.77 (1.47–2.12)
Customer service/Social services	1.30 (0.27–3.81)	3	2301.4	0.11 (0.04–0.35)
Healthcare professional	22.59 (12.91–36.69)	16	708.2	1.88 (1.13–3.14)
**Years of exposure**				
<1 year	11.69 (10.18–13.37)	213	18,216.3	1.00 (Ref.)
1 to <5 years	15.63 (12.66–19.09)	96	6141.6	1.85 (1.45–2.36)
5 to <10 years	25.66 (20.12–32.27)	73	2844.4	2.92 (2.23–3.82)
≥10 years	37.11 (31.58–43.33)	160	4311.5	3.61 (2.92–4.46)
**Age in years**				
Per 5-year increase				1.08 (1.04–1.12)
**Incidence by quartiles**				
Q1: 18–28	12.91 (10.57–15.61)	106	8210.6	—
Q2: >28–36	16.66 (13.94–19.76)	132	7922.5	—
Q3: >36–45	16.61 (13.83–19.79)	125	7524.2	—
Q4: >45–76	22.78 (19.57–26.38)	179	7856.5	—
**Abdominal waist**				
Per 5 cm increase				0.99 (0.95–1.04)
**Incidence by quartiles**				
Q1: 53–84	20.17 (16.92–23.86)	136	6742.1	—
Q2: >84–91	19.73 (16.46–23.46)	128	6487.5	—
Q3: >91–98	15.84 (12.84–19.32)	97	6124.2	—
Q4: >98–147	16.50 (13.32–20.22)	93	5635.7	—
**Systolic blood pressure**				
Per 5 mmHg increase				1.03 (1.00–1.07)
**Incidence by quartiles**				
Q1: 83–103	14.01 (12.08–16.16)	188	13,419.5	—
Q2: >103–113	19.23 (16.39–22.42)	163	8475.9	—
Q3: >113–123	20.70 (17.52–24.29)	150	7245.1	—
Q4: >123–202	17.17 (12.27–23.39)	40	2329.0	—
**Diastolic blood pressure**				
Per 5 mmHg increase				1.02 (0.99–1.04)
**Incidence by quartiles**				
Q1: 53–63	15.72 (13.61–18.07)	198	12,591.7	—
Q2: >63–73	15.65 (13.33–18.27)	161	10,284.4	—
Q3: >73–83	23.03 (19.65–26.82)	165	7166.0	—
Q4: >83–123	12.04 (7.02–19.28)	17	1411.7	—
**Glucose**				
Per 5 mg/dL increase				1.03 (1.02–1.05)
**Incidence by quartiles**				
Q1: 56–83.1	9.39 (7.32–11.86)	70	7454.0	—
Q2: >83.1–91	12.82 (10.25–15.83)	86	6709.6	—
Q3: >91–98	14.24 (11.66–17.23)	106	7441.6	—
Q4: >98–377.7	16.93 (13.49–20.99)	83	4901.2	—
**Triglycerides**				
Per 5 mg/dL increase				1.01 (1.00–1.02)
**Incidence by quartiles**				
Q1: 27–89	12.88 (9.73–16.73)	56	4347.3	—
Q2: >89–124	12.20 (9.11–16.00)	52	4261.2	—
Q3: >124–178	17.25 (13.50–21.73)	72	4173.1	—
Q4: >178–957	18.13 (14.29–22.70)	76	4191.2	—
**Total cholesterol**				
Per 5 mg/dL increase				1.03 (1.01–1.04)
**Incidence by quartiles**				
Q1: 79–167	11.88 (8.90–15.54)	53	4462.2	—
Q2: >167–191	14.44 (11.05–18.55)	61	4224.1	—
Q3: >191–219.45	17.08 (13.34–21.54)	71	4157.1	—
Q4: >219.45–456.62	17.11 (13.36–21.58)	71	4150.5	—

Note: PY = person-years. Incidence rates are per 1000 PY. HR = hazard ratio; CI = confidence interval; Ref. = reference category.

## Data Availability

All relevant data are within the manuscript and its Supporting Information files.

## Supplementary Materials

The following supporting information can be downloaded at: https://www.mdpi.com/article/10.3390/jcm15197650/s1, Table S1: STROBE checklist for cohort studies; Table S2: Age- and sex-standardized incidence rates by occupational category and years of occupational exposure.
