# Supplementary material for: Incidence and Demographic, Clinical, and Occupational Patterns of Dental Caries and Gingivitis in Workers: A Retrospective Descriptive Cohort Study
Source: J Clin Med. 2026 Oct 2;15(19):7650. doi: 10.3390/jcm15197650 (PMC13665839; doi:10.3390/jcm15197650)
Supplement: Supplementary file 1 [file jcm-15-07650-s001.zip › jcm-4351386-supplementary.pdf]

Supplementary Material

Supplementary Table S1. STROBE checklist for cohort studies.

STROBE No.	STROBE item	Compliance	Evidence in revised manuscript	Location
1a	Title: indicate the study design with a common term in the title or abstract	Yes	The revised title and abstract identify the study as a retrospective descriptive cohort study.	Title; Abstract
1b	Abstract: provide an informative and balanced summary of what was done and found	Yes	The revised abstract reports objective, design, sample size, corrected incidence rates per 1,000 person-years, crude HRs, and cautious conclusions.	Abstract
2	Background/justification: explain the scientific basis and justification	Yes	The Introduction now clarifies occupational, behavioral, access-to-care, and cardiometabolic pathways linking work and oral health.	Introduction
3	Objectives: state specific objectives and any prespecified hypotheses	Yes	The revised objective estimates incidence and describes demographic, clinical, behavioral, and occupational differences without causal claims.	Introduction
4	Study design: present key elements of the design early in the article	Yes	A retrospective descriptive cohort design is specified, with comparative estimates interpreted as crude descriptive contrasts.	Methods: Type and design
5	Setting: describe setting, locations, and relevant dates	Yes	Occupational clinic records from 2014 to 2021; routine annual or biennial occupational evaluations.	Methods: Population and sample; Procedures
6a	Participants: eligibility criteria, sources and methods of selection; follow-up methods	Yes	Workers with at least two dental evaluations separated by at least one year and complete essential records were included; outcome-specific event-free subcohorts were constructed.	Methods: Selection criteria; Follow-up
6b	Matched studies: matching criteria and number of exposed/unexposed	Not applicable	Not a matched study.	-
7	Variables: clearly define outcomes, exposures, predictors, confounders, and modifiers; diagnostic criteria	Yes	Caries and gingivitis screening definitions were expanded; early non-cavitated lesions, severity indices, and radiographic lesions were not systematically recorded.	Methods: Variables and measurement
8	Data sources/measurement: sources and methods of measurement	Yes	Routine occupational dental examination, anthropometry, fasting biochemistry, blood pressure measurement, and occupational questionnaire procedures are described.	Methods: Procedures
9	Biases: describe efforts to address potential sources of bias	Yes	The revised limitations discuss selection by valid follow-up, interval censoring, diagnostic variability, residual confounding, and lack of company/industry identifiers.	Discussion: Limitations
10	Study size: explain how it was determined	Yes	All eligible records were included; precision was evaluated using event counts and Poisson confidence intervals.	Methods: Statistical accuracy
11	Quantitative variables: how they were handled in the analysis; groupings and why	Yes	Continuous variables were analyzed per 5-unit increment and categorized into quartiles only for descriptive incidence patterns.	Methods: Statistical analysis
12a	Statistical methods: describe all methods, including those for controlling confounding	Yes	Incidence rates were corrected to person-years; crude Cox models were used as descriptive time-to-event contrasts; age- and sex-standardized rates were added as supplementary descriptive analyses.	Methods: Statistical analysis
12b	Methods for subgroups and interactions	Not applicable	No interaction analyses were planned because the study was descriptive.	Methods: Statistical analysis
12c	Missing data: how it was addressed	Partial	Eligibility required complete essential records; missingness by nonessential variables was not the primary target of this secondary analysis.	Methods: Selection criteria; Limitations
12d	Losses to follow-up: how they were addressed	Yes	Workers without valid follow-up were quantified in the flow chart, and selection due to repeated evaluations is discussed as a limitation.	Results: Figure S1; Discussion: Limitations
12e	Sensitivity analyses	Not applicable	No sensitivity analyses were conducted; standardized descriptive rates were added as supplementary magnitude measures, not sensitivity analyses.	Supplementary Table S5
13a	Participants: numbers at each stage	Yes	79,660 evaluated; 12,049 with valid follow-up; outcome-specific at-risk cohorts and baseline exclusions reported.	Results; Figure S1
13b	Reasons for non-participation at each stage	Partial	Primary exclusion was lack of valid follow-up; additional exclusions were baseline caries, gingivitis, or either event depending on outcome.	Results; Figure S1
13c	Use of flowchart	Yes	A flow chart of participant selection with corrected incidence rates is provided.	Figure S1
14a	Descriptive data: characteristics of participants and potential confounders	Yes	Demographic, occupational, behavioral, clinical, biochemical, and baseline oral health characteristics are reported.	Supplementary Table S1
14b	Number of participants with missing data per variable	Partial	Only complete essential records were included; variable-specific missingness is not separately tabulated.	Methods: Selection criteria
14c	Summarize follow-up time	Yes	Median follow-up time and number of evaluations are now reported; corrected person-years are shown in incidence tables.	Results; Supplementary Tables S2-S4
15	Outcome data: numbers of events or summary measures over time	Yes	Events, corrected person-years, corrected incidence rates, and 95% CIs are reported for caries, gingivitis, and combined outcome.	Supplementary Tables S2-S4
16a	Main results: crude estimates and, where appropriate, adjusted estimates with precision	Yes	Crude HRs with 95% CIs are reported and interpreted as descriptive contrasts, not causal effects.	Results; Supplementary Tables S2-S4
16b	Category limits when continuous variables were categorized	Yes	Quartile limits were reformatted for readability.	Supplementary Tables S2-S4
16c	Translation to absolute risks for a meaningful time period	Partial	Corrected incidence rates, rate differences, and standardized rate ratios are provided as absolute and relative magnitude measures.	Results; Supplementary Table S5
17	Other analyses	Yes	Age- and sex-standardized incidence rates with approximate 95% CIs, standardized rate differences, and standardized rate ratios were added.	Supplementary Table S5
18	Key results	Yes	The revised discussion summarizes corrected incidence rates, crude descriptive contrasts, and standardized-rate interpretation.	Discussion: Main findings
19	Limitations	Yes	The revised limitations address pragmatic diagnostic definitions, no formal calibration, interval censoring, selection by follow-up, residual confounding, no company clustering, and no industry codes.	Discussion: Limitations
20	Interpretation	Yes	Interpretation was harmonized with the descriptive design and avoids causal or etiologic language.	Discussion
21	Generalizability	Yes	The findings are interpreted primarily for workers undergoing occupational health evaluations in the source clinic; industry representativeness could not be assessed.	Discussion: Limitations
22	Funding	Yes	Funding/APC statement is reported in the manuscript.	Funding statement

Note: Rows highlighted in yellow indicate checklist entries that were updated to reflect the revised manuscript, corrected person-time, additional descriptive analyses, or harmonized terminology.

Supplementary Table S2. Age- and sex-standardized incidence rates by occupational category and years of occupational exposure.

Panel A. Caries by occupational category

Characteristic	At-risk n	Events	Crude rate	Standardized rate (95% CI)	Std. rate difference	Std. rate ratio
Office/administrative	4,304	141	11.62	11.70 (9.73-13.67)	0.00	1.00
Manual/physical	6,486	311	18.87	18.71 (16.55-20.88)	7.01	1.60
Customer service/social services	818	3	1.30	2.34 (0.00-5.62)	-9.36	0.20
Healthcare professional	280	15	21.10	21.80 (10.63-32.97)	10.10	1.86

Panel B. Caries by years of occupational exposure

Characteristic	At-risk n	Events	Crude rate	Standardized rate (95% CI)	Std. rate difference	Std. rate ratio
<1 year	6,830	186	10.16	9.77 (8.28-11.25)	0.00	1.00
1 to <5 years	2,460	78	12.67	12.41 (9.61-15.22)	2.65	1.27
5 to <10 years	1,097	69	24.16	24.37 (16.92-31.83)	14.61	2.50
≥10 years	1,501	137	31.78	27.67 (21.28-34.07)	17.90	2.83

Panel C. Gingivitis by occupational category

Characteristic	At-risk n	Events	Crude rate	Standardized rate (95% CI)	Std. rate difference	Std. rate ratio
Office/administrative	4,340	58	4.74	4.71 (3.47-5.95)	0.00	1.00
Manual/physical	6,541	85	5.12	4.84 (3.80-5.89)	0.13	1.03
Customer service/social services	823	1	0.43	0.37 (0.00-1.10)	-4.34	0.08
Healthcare professional	280	5	7.04	7.82 (0.86-14.79)	3.11	1.66

Panel D. Gingivitis by years of occupational exposure

Characteristic	At-risk n	Events	Crude rate	Standardized rate (95% CI)	Std. rate difference	Std. rate ratio
<1 year	6,892	61	3.30	3.05 (2.25-3.85)	0.00	1.00
1 to <5 years	2,479	35	5.63	5.57 (3.69-7.46)	2.52	1.83
5 to <10 years	1,102	15	5.24	6.23 (1.89-10.57)	3.18	2.04
≥10 years	1,511	38	8.74	7.46 (4.19-10.74)	4.41	2.45

Approximate 95% confidence intervals (95% CIs) for directly standardized rates were estimated using a Poisson normal approximation within age-sex strata. Standardized rate differences and ratios were used as descriptive measures of magnitude and were not interpreted as causal estimates. The standardized rates in S5 were checked against the values described in the main text and are fully consistent with those results.
